# Neuro-immune and metabolic disorders in association with depression, anxiety, and chronic fatigue-fibromyalgia symptoms due to nonalcoholic fatty liver disease

**DOI:** 10.1017/S1092852926100960

**Published:** 2026-05-26

**Authors:** Walaa Abdulhussein Al-Azzawi, Hamid Yaghooti, Hussein Al-Hakeim, Michael Maes

**Affiliations:** 1 Department of Clinical Biochemistry, Faculty of Medical Sciences, Tarbiat Modares University Faculty of Medical Sciences, Islamic Republic of Iran; 2 Department of Chemistry, Faculty of Science, University of Kufa, Iraq; 3 Sichuan Provincial Center for Mental Health, Sichuan Provincial People’s Hospital, School of Medicine, University of Electronic Science and Technology of China, Chengdu 610072, China; 4 Key Laboratory of Psychosomatic Medicine, Chinese Academy of Medical Sciences, Chengdu, 610072, China; 5 Department of Psychiatry, Faculty of Medicine, Chulalongkorn University, Bangkok, Thailand; 6 Department of Psychiatry, Medical University of Plovdiv, Plovdiv, Bulgaria; 7 Research Institute, Medical University of Plovdiv, Plovdiv, Bulgaria; 8 Kyung Hee University, Seoul, Dongdaemun-gu, South Korea

**Keywords:** Depression, anxiety, chronic fatigue syndrome, neuroimmune, metabolism, tryptophan, atherogenicity

## Abstract

**Background:**

Nonalcoholic fatty liver disease (NAFLD) is frequently associated with depression, anxiety, and chronic fatigue syndrome (CFS). Major depression and NAFLD are accompanied by low-grade inflammation, increased atherogenicity, and insulin resistance. There is a paucity of data on serum tryptophan and tryptophan catabolites (TRYCATs) in relation to these symptoms in patients with NAFLD. This study aims to determine the relationships between the severity of NAFLD, the severity of the above neuropsychiatric symptoms and neuro-immune, metabolic, and TRYCAT pathway biomarkers.

**Methods:**

This case–control study included 56 patients with Grade 1 NAFLD, 52 with Grade 2 NAFLD, and 60 healthy controls. We assessed serum insulin, tryptophan, kynurenine, 3-hydroxykynurenine, kynurenic acid, indoleamine 2,3-dioxygenase 1 (IDO1), interleukin (IL)-6, IL-10, HOMA2 insulin resistance index (HOMA2IR), and atherogenic *indices.*

**Results:**

The severity of depression, anxiety, and fibro-fatigue symptoms was significantly higher in NAFLD than in controls and in grade 2 as compared with grade 1 NAFLD. The atherogenic and HOMA2IR indices increased significantly from controls to grade 1 and then to grade 2. Serum tryptophan, kynurenine, kynurenic acid, and 3-OH-kynurenine were significantly lower in NAFLD and especially in grade 2 than in controls, while IL-6 and IL-10 were higher in grade 2 NAFLD than in controls. The rating scale scores were significantly and positively correlated with liver tests, atherogenicity, and HOMA2 indices, and inversely with tryptophan and TRYCATs.

**Conclusions:**

Affective and fibro-fatigue symptoms due to NAFLD might be mediated by the cumulative neurotoxic effects of lipids, IR, and lowered anti-inflammatory and antioxidant effects of the TRYCAT pathway.

## Graphical abstract

**Figure:**
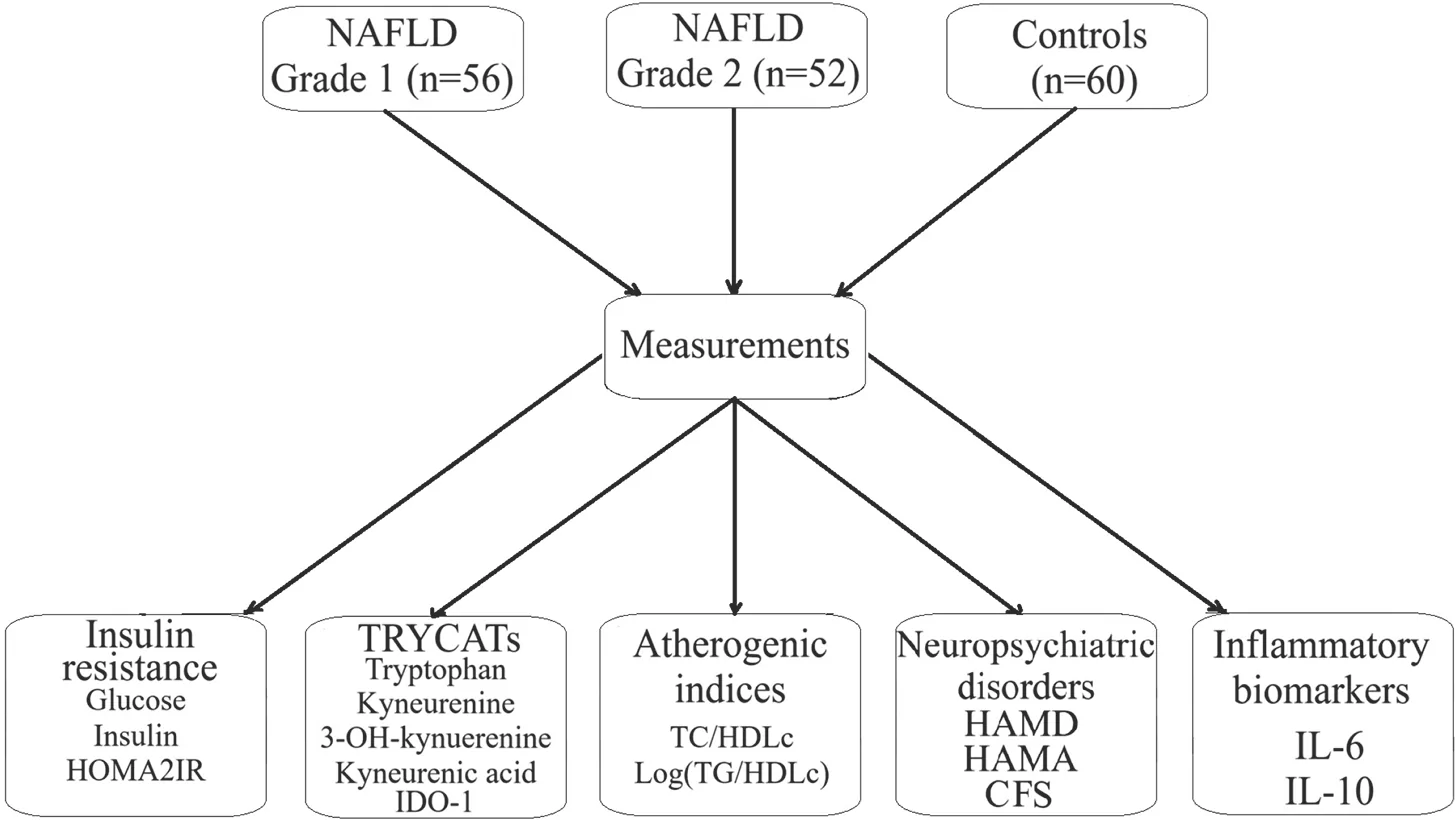

## Highlights

- The severity of depression, anxiety, and fatigue was increased in NAFLD patients.
- Grade 2 NAFLD patients have higher symptoms than Grade 1.
- Lower tryptophan catabolites worsen these affective and physiosomatic symptoms.
- Atherogenicity and insulin resistance worsen neuropsychiatric symptoms.
- Lower tryptophan is another target to treat affective and physiosomatic symptoms.

## Introduction

Nonalcoholic fatty liver disease (NAFLD) results from the accumulation of fat in the liver (hepatic steatosis), which can be evidenced by liver imaging and histological assessments.^1^ This adipose tissue accumulation is not attributable to alcoholism, substance abuse, or other medical conditions. NAFLD severity is classified into distinct classes based on histologic characteristics: grade 1: steatosis, grade 2: steatosis accompanied by lobular inflammation and inflated hepatocytes, and grade 3: steatosis, lobular inflammation, ballooned hepatocytes, and the presence of Mallory hyaline or fibrosis.^2^ NAFLD is associated with the potential progression to more severe forms, such as NASH, along with liver complications, such as fibrosis, cirrhosis, and liver cancer, and extrahepatic complications, such as cardiovascular diseases, type 2 diabetes mellitus, and renal dysfunctions.^3^^,^^4^

NAFLD is recognized as a hepatic manifestation of metabolic syndrome, associated with obesity, insulin resistance (IR), atherogenic dyslipidemia, advanced atherosclerosis, and proinflammatory mediators produced by the steatotic or inflamed liver.^5^^–^^8^ Intertwined interactions between these factors characterize NAFLD. For example, hyperinsulinemia may induce hepatic lipogenesis and fat accumulation in the liver,^9^ while IR is linked to a heightened risk of fatty liver disease.^10^ Reduced high-density lipoprotein cholesterol (HDLc) levels correlate with diminished tissue cholesterol efflux and impaired antioxidant capabilities, influencing the development of NAFLD.^11^ Increased low-density lipoprotein cholesterol (LDLc) has been demonstrated in previous research to be an important indicator of NAFLD.^12^

Numerous reports indicate that NAFLD may be linked to chronic low-grade hepatic inflammation.^13^^,^^14^ Immunological mediators, including interleukin (IL)-6, a proinflammatory cytokine, and IL-10, an immunoregulatory cytokine, play a crucial role in alcoholic liver injury and inflammation.^15^ Moreover, C-reactive protein, an acute phase protein, and tumor necrosis factor (TNF)-*α*, a proinflammatory cytokine, are markedly elevated in individuals with NAFLD, particularly in those with grade 2 NAFLD.^16^ Complex interactions between elevated insulin resistance, atherogenicity, and immunological activation (e.g., C-reactive protein [CRP] levels) correlate with a heightened risk of NAFLD.^17^ Low-grade inflammation and lipid buildup may precipitate insulin resistance in NAFLD.^17^^,^^18^

Neuropsychiatric disorders, such as chronic fatigue syndrome (CFS), depression, and anxiety, are more common in individuals with NAFLD than in the general population.^19^^–^^21^ Chronic fatigue is a prevalent clinical symptom of NAFLD and is regarded as an independent indicator of poorer patient-reported outcomes.^22^^–^^25^ A meta-analysis indicated that persons with NAFLD had a significantly elevated risk of depression.^20^ Anxiety is a common affective symptom associated with NAFLD that might also influence overall health-related quality of life.^26^^,^^27^

The activation of immune-inflammatory pathways and disruptions in metabolic pathways, including heightened atherogenicity, are characteristic features of affective disorders and fibro-fatigue symptoms.^28^^–^^31^ The activation of these pathways is believed to contribute to the onset of CFS and affective symptoms.^28^^,^^32^ Elevated levels of IL-6 and IL-10 have been identified as biomarkers for major depression, with notable relationships with the severity of depression ratings.^33^^–^^35^

Similarly, diminished HDLc, elevated triglyceride and LDLc levels, and heightened atherogenicity indices, such as the Castelli risk index 1 (CRI1) and the atherogenic index of plasma (AIP), are commonly observed in individuals exhibiting heightened affective and CFS symptomatology.^36^ Increased IR impacts immune and atherogenicity pathways and aggravates the severity of affective and fibro-fatigue symptoms.^36^ Consequently, one may postulate that interactions between these pathways could be linked to the emergence of affective and fibro-fatigue symptoms resulting from NAFLD.

The activation of immune-inflammatory pathways may trigger the tryptophan catabolite (TRYCAT) pathway, leading to decreased serum L-tryptophan and elevated serum levels of TRYCATs, including kynurenine (KYN), 3-hydroxy-kynurenine (3HK), and kynurenic acid (KA).^37^ This pathway is activated by indoleamine 2,3-dioxygenase 1 (IDO1), which is prompted by inflammatory cytokines such as interferons and M1 macrophage cytokines, or by tryptophan-2,3-dioxygenase (TDO) in the liver, which is stimulated by glucocorticoids, typically elevated during immunological activation.^38^ In nonalcoholic hepatic steatosis, the TRYCAT pathway is activated by gut-derived inflammation.^39^ Reduced levels of L-tryptophan are a persistent indicator of depression and anxiety, whereas elevated TRYCAT levels, including KYN and IDO1 activation, may manifest in severe forms of major depression.^40^ Moreover, heightened atherogenicity may correlate with diminished tryptophan levels and an elevated KYN/tryptophan ratio, suggesting activation of IDO1 or TDO.^41^ However, no data indicate whether in NAFLD, the severity of chronic fatigue syndrome and affective symptoms correlates with activation of the TRYCAT pathway, as evidenced by reduced tryptophan levels and elevated TRYCATs.

The current study seeks to investigate (a) whether serum tryptophan levels are diminished and TRYCATs are elevated in NAFLD, particularly in grade 2 NAFLD, relative to controls; and (b) whether serum concentrations of tryptophan and TRYCATs influence the severity of fibro-fatigue and affective symptoms due to NAFLD, independently of atherogenic and IR indices.

## Participants and methods

### Participants

In the present case–control study, we included 56 grade 1 NAFLD patients and 52 Grade 2 NAFLD patients in addition to 60 healthy controls. Participants included individuals of both sexes (55 females and 113 males), aged 22–69 years. Participants were recruited from the Departments of Internal Medicine at Al-Kadhimiya and Al-Kindi Medical City from September 2024 until December 2024. Visual hepatic echogenicity was carried out to evaluate liver fat content and liver damage.^42^ Hepatic echogenicity was also used to grade the severity of NAFLD into grade 1, that is, slightly increased echogenicity with normal visualization of the diaphragm and intrahepatic vessel borders. Grade 2 (moderate NAFLD) is defined as moderately increased echogenicity with slightly impaired visualization of the diaphragm or intrahepatic vessels.^43^ We did not include patients with grade 3 NAFLD. All controls had a normal liver echogenicity.

The present study excluded patients and controls who had type 1 diabetes, systemic (auto)immune diseases like inflammatory bowel disease, rheumatoid arthritis, renal disease, as well as neurodegenerative and neuroinflammatory disorders, including Parkinson’s and Alzheimer’s disease, multiple sclerosis, or stroke. Also, we excluded any subjects with a lifetime or current history of psychiatric axis-1 disorders, such as major affective disorders (major depressive disorder and bipolar disorder, dysthymia, generalized anxiety disorder, panic disorder), schizoaffective disorder, schizophrenia, psycho-organic syndrome, substance-use disorders (except tobacco-use disorder), CFS, and fibromyalgia.

Moreover, according to the requirements of the homeostatic model assessment (HOMA) calculator software, the following patient exclusion criteria were applied: patients with obvious serious overt diabetes complications, such as heart disease, liver diseases, or renal disease; and patients with fasting insulin >400 pM. Additionally, since metformin may affect insulin sensitivity and IR,^44^ we excluded individuals taking metformin.^45^ Women who were pregnant or nursing were also excluded from this research.

Before participating in the study, all controls and patients, or their parents or legal guardians, supplied written informed consent. The Najaf Health Directorate, Training, and the Human Development Center (Document No. 20147/2024), and the institutional ethics boards of the University of Kufa approved the study. Our institutional review board follows the International Guideline for Human Research Safety, and the study was carried out following Iraqi and international ethical and privacy laws, such as the World Medical Association’s Declaration of Helsinki, The Belmont Report, the CIOMS Guideline, and the International Conference on Harmonization of Good Clinical Practice (ICH-GCP).

### Clinical severity measurements

A semi-structured interview was conducted by a senior psychiatrist specializing in affective disorders to collect clinical and sociodemographic data from both patients and controls. The severity of fibro-fatigue symptoms was assessed by a senior psychiatrist using the Fibro-Fatigue scale.^46^ The level of anxiety was evaluated using the Hamilton Anxiety Rating Scale (HAMA).^47^ Hamilton’s Depression Rating Scale (HAMD)^48^ was completed by the same psychiatrist to measure the severity of depression. Since both the HAMD and HAMA scales comprise many physiosomatic (formerly known as psychosomatic items), which may interfere with the interpretation of depressive and anxiety symptoms in a medical illness, we have computed the sum of the key HAMD depressive symptoms (sum of sad mood + feelings of guilt + suicidal thoughts + loss of interest, labeled as “pure depression”). Likewise, we have computed the sum of the key HAMA anxiety symptoms (sum of anxious mood + tension + fears + anxiety behavior during the interview, labeled as “pure anxiety”). In order to more precisely assess the physiosomatic symptoms of the FF, namely, muscular pain, muscle tension, fatigue, autonomous symptoms, gastrointestinal symptoms (GIS), headache, and a flu-like malaise (labeled “pure FF”), we computed the sum of all items without items reflecting sadness, cognitive symptoms, insomnia, and irritability. A comprehensive index of all the physiosomatic symptoms of the HAMD, HAMA, and FF scores was computed as the sum of the *z* scores of all physiosomatic HAMD (somatic anxiety + GIS anxiety + genitourinary + hypochondriasis), HAMA (somatic sensory + cardiovascular + GIS + genitourinary + autonomic symptoms), and physiosomatic FF symptoms (muscular pain + muscle tension + fatigue + autonomous symptoms + GIS symptoms + headache + a flu-like malaise).

### Assessments of biomarkers

Fasting blood samples (12–14 hours) were collected in the early morning between 7.30 and 9 a.m., before having breakfast. Five milliliters of venous blood were extracted and transferred to clean tubes. Hemolyzed specimens were discarded. After 15 minutes, the blood samples were centrifuged at 1200 × g for 5 minutes to separate the serum, which was then transferred to three fresh Eppendorf tubes for testing. Fasting blood glucose (FBG), triglycerides (*TG), total cholesterol (TC), high-density lipoprotein cholesterol (HDLc), alanine aminotransferase (ALT), aspartate aminotransferase (AST), and alkaline phosphatase (ALP)* levels were tested spectrophotometrically using kits provided by Biolabo® (Maizy, France). A commercial ELISA sandwich kit was used to measure serum insulin (DRG® International Inc., USA). The sensitivity of the insulin assay was 1.76 μIU/ml (12.22 pM), and the CV% was 2.6%. Tryptophan, KYN, 3-HK, KA, IDO1, IL-6, and IL-10 were measured using ELISA kits supplied by Nanjing Pars Biochem Co., Ltd. (Nanjing, China). All ELISA kits have intra-assay CVs below 10.0%. Samples with high analyte concentrations were diluted.

We computed three different atherogenicity indices, namely Castelli risk index 1 (TC/HDLc) and atherogenicity index of plasma (API) (triglycerides/HDLc).^49^^,^^50^ The Homeostasis Model Assessment 2 (HOMA2) calculator© (Diabetes Trials Unit, University of Oxford; https://www.dtu.ox.ac.uk/homacalculator/download.php) was used to calculate the IR index (HOMA2-IR) using fasting serum insulin and glucose levels. Furthermore, we computed two new *z*-unit-based composite scores: a first reflecting liver integrity, as zALP + zAST + zALT, and a second reflecting the TRYCAT pathway, computed as zTRP + zKYN + z3HK + zKA.

### Statistical analysis

A one-way analysis of variance was used to compare scale variables across groups, while contingency tables (*χ*^2^ tests) were used to assess the associations between categorical variables. Pearson’s product–moment correlation, Spearman’s rank-order correlation coefficients, and partial correlation coefficients were used to analyze the relationships among scale variables. We conducted a binary logistic regression analysis with NAFLD diagnosis as the dependent variable (and controls as the reference group) and biomarkers as explanatory variables. The odds ratio with 95% confidence intervals was computed, as well as the accuracy of the prediction with sensitivity and specificity. Multiple regression analysis was used to delineate the most important biomarkers predicting the neuropsychiatric symptom domains using manual and stepwise automatic (*p*-to-entry of 0.05 and *p*-to-remove of 0.06) approaches. We determined the standardized beta coefficients for each significant explanatory variable using t-statistics with accurate *p*-values and the model F-statistics with total variance explained (*R*^2^), which was used as the effect size. Furthermore, the analysis was checked for homoscedasticity (using the White and Breusch-Pagan tests) and collinearity issues (using VIF and tolerance).

We employed multilayer perceptron Neural Network (NN) models to examine the effects of biomarkers (entered as input variables) predicting the neuropsychiatric scores (entered as output variables). We used an automated feedforward architecture model to train the network with two hidden layers of up to 8 nodes employing minibatch training with gradient descent and a maximum of 250 epochs. The stopping rule was one consecutive step with no further decrease in the error term. The study group was split into three sets, namely, a training set to estimate network parameters (46.67% of all participants), a testing set to prevent overtraining (20.0%), and a holdout set to evaluate the final network (33.33%). Error, relative error, and importance and relative importance of all input variables were computed. Model adequacy was assessed using accuracy and the area under the ROC curve (AUC). All statistical analyses were conducted using IBM SPSS Statistics version 29 for Windows.

The a priori estimated sample size was calculated using G*Power 3.1.9.7. Multiple regression analyses utilizing the phenome scores as the dependent variables served as the primary outcome measures. Utilizing an effect size of 0.25, the estimated sample size was 69 participants, assuming a power of 0.8, an alpha error probability of 0.05, and inclusion of up to 8 covariates. The post hoc statistical power calculated for the regressions employing pure depression or physiosomatic symptoms as dependent variables demonstrated a power of 1.

## Results

### Demographic and clinical data

The results in Table 1 show the demographic, clinical, and routine biochemical results of Grade 1 and 2 NAFLD patients as compared with the healthy control groups. There were no statistically significant differences in age, sex, smoking status, BMI, or marital status among the three study groups. The unemployment rate was significantly higher in NAFLD than in controls. Table 1 indicates that scores of pure depression, pure anxiety, pure FF, and physiosomatic scores were significantly different among the three groups and increased from controls to grade 1 to grade 2 NAFLD.

**Table 1.** Demographic and Clinical Characteristics of Patients with Grade 1 and Grade 2 Non-Alcoholic Liver Disease (NAFLD) and Healthy Controls (HC) Table 1. long description.

Parameters	HC (*n* = 60)	Grade 1 (*n* = 56)	Grade 2 (*n* = 52)	*F*/*χ*^2^	df	*p*
Age (years)	50.7 ± 5.5	49.8 ± 7.8	50.37 ± 8.57	0.26	2/165	0.775
Sex (F/M)	18/42	24/32	13/39	4.22	2	0.125
Smoking (no/yes)	11/49	7/49	5/47	1.89	2	0.405
Marriage (no/yes)	21/39	17/39	10/42	3.53	2	0.174
Employment (no/yes)	11/49	38/18	32/20	33.81	2	<0.001
Body mass index (kg/m^2^)	27.86 ± 3.79	28.17 ± 4.36	28.31 ± 3.66	0.20	2/165	0.819
Pure depression score	1.2 ± 0.9 ^B,C^	3.0 ± 1.5 ^A,C^	4.4 ± 1.5 ^A,B^	86.90	2/165	<0.001
Pure anxiety score	2.1 ± 1.0 ^B,C^	4.5 ± 2.5 ^A,C^	6.4 ± 2.1 ^A,B^	69.74	2/165	<0.001
Pure fibro fatigue score	5.0 ± 1.7 ^B,C^	12.0 ± 3.8 ^A,C^	13.7 ± 5.0 ^A,B^	89.39	2/165	<0.001
Physiosomatic score (z scores)	−1.143 ± 0.279 ^B,C^	0.350 ± 0.503 ^A,C^	0.942 ± 0.580 ^A,B^	304.29	2/165	<0.001
Total serum bilirubin (mg/dL)	0.815 ± 0.166	0.768 ± 0.174	0.802 ± 0.188	1.08	2/165	0.342
Direct serum bilirubin (mg/dL)	0.232 ± 0.068	0.248 ± 0.061	0.243 ± 0.062	1.03	2/165	0.358
Indirect serum bilirubin (mg/dL)	0.583 ± 0.160	0.52 ± 0.175	0.559 ± 0.209	1.80	2/165	0.169
Alkaline phosphatase (ALP) (IU/L)	97.30 ± 16.48 ^B,C^	117.68 ± 17.12 ^A^	121.46 ± 20.93 ^A^	29.39	2/165	<0.001
Aspartate transferase (AST) (IU/L)	21.53 ± 8.31 ^C^	23.07 ± 8.18 ^C^	28.15 ± 8.16 ^A,B^	9.69	2/165	<0.001
Alanine aminotransferase (ALT) (IU/L)	19.07 ± 6.71 ^C^	23.35 ± 7.13 ^C^	24.76 ± 1.24 ^A,B^	8.68	2/165	<0.001
AST/ALT ratio	1.33 ± 0.78	1.15 ± 0.67	1.34 ± 0.68	1.23	2/165	0.294
zALP + zAST + zALT (z score)	−0.740 ± 0.769 ^B,C^	0.166 ± 0.707 ^A,C^	0.674 ± 0.956 ^B,C^	43.91	2/165	<0.001

Results expressed as mean ± standard deviation and analyzed by ANOVA. A, B, C: results of pairwise group comparisons. Binomial data were expressed as ratios and analyzed by the chi-squared test, F/χ2: F-statistic value for continuous variables or chi-square statistic value for categorical variables, df: degree of freedom between groups/within groups, p: probability value.

Total bilirubin, direct bilirubin, indirect bilirubin, and the AST/ALT ratio were not significantly different among the three study groups. ALP was significantly higher in NAFLD than in controls, whereas AST and ALP were higher in grade 2 than in grade 1 NAFLD and healthy controls. The composite zALP + zAST + zALT score was significantly different between the three groups and increased from controls to grade 1 to grade 2 NAFLD.

### Biomarkers

The measurements of the biomarkers in the three study groups are presented in Table 2. Tryptophan and the zTRP + zKYN + z3HK + zKA score significantly differed among the three study groups, decreasing from controls to grade 1 to grade 2. KYN, KA, and 3HK were significantly lower in grade 2 than in controls, and KYN and KA were significantly lower in grade 2 than in grade 1. There were no significant differences between the IDO1 and KMO indices between the three groups. IDO1 concentrations, TC, LDLc, Castelli risk index 1, AIP, glucose, and the HOMA2IR index were significantly different between the three groups and increased from controls to grade 1 to grade 2. IL-6 was significantly higher in grade 2 than in controls, while patients with grade 1 took up an intermediate position. IL-10 and triglycerides were significantly higher in NAFLD than in controls, while HDLc was lower in grade 2 than in the two other groups. Insulin was higher in grade 2 than in the other two groups.

**Table 2.** Comparison of the Serum Levels of the Measured Biomarkers among Patients with Grade 1 and Grade 2 Non-Alcoholic Liver Disease (NAFLD) and Healthy Controls (HC) Table 2. long description.

Parameters		HC (*n* = 60)	Grade 1 (*n* = 56)	Grade 2 (*n* = 52)	F	df	*p*
L-Tryptophan (TRP)	(μM)	62.38 (2.98) ^B,C^	51.82 (3.1) ^A,C^	41.88 (3.22) ^A,B^	11.00	2/162	<0.001
Kynurenine (KYN)	(μM)	2.34 (0.10) ^C^	2.11 (0.11) ^C^	1.78 (0.11) ^A,B^	6.69	2/162	0.002
3-Hydroxy-kynurenine (3HK)	(μM)	19.74 (1.08) ^C^	17.68 (1.12)	14.64 (1.16) ^A^	7.18	2/162	0.001
Kynurenic acid (KA)	(μM)	35.63 (1.35) ^C^	33.84 (1.41) ^C^	24.33 (1.45) ^A,B^	18.19	2/162	<0.001
KYN/TRP (IDO index)		0.048 (0.004)	0.047 (0.004)	0.053 (0.004)	0.50	2/162	0.607
KA/KYN (KAT index)		16.67 (1.27)	19.67 (1.33)	16.24 (1.37)	1.95	2/162	0.146
3HK/KYN (KMO index)		9.34 (0.88)	10.01 (0.92)	9.66 (0.95)	0.13	2/162	0.874
IDO1	(ng/ml)	19.31 (1.25) ^B,C^	26.21 (1.30) ^A,C^	31.95 (1.34) ^A,B^	24.09	2/162	<0.001
zTRP + zKYN + z3HK + zKA (z score)		0.579 (0.109) ^B,C^	0.071 (0.114) ^A,C^	−0.744 (0.117) ^A,B^	34.42	2/162	<0.001
Interleukin–6	(pg/ml)	5.44 (0.53) ^C^	6.68 (0.55)	7.58 (0.57) ^A^	5.08	2/162	0.007
Interleukin–10	(pg/ml)	8.72 (1.38) ^B,C^	15.19 (1.44) ^A^	17.26 (1.49) ^A^	11.84	2/162	<0.001
Triglycerides (TG)	(mM)	1.17 (0.05) ^B,C^	1.58 (0.05) ^A^	1.70 (0.05) ^A^	35.50	2/162	<0.001
Total cholesterol (TC)	(mM)	4.78 (0.06) ^B,C^	5.22 (0.07) ^A,C^	5.59 (0.07) ^A,B^	37.67	2/162	<0.001
High-density lipoprotein (HDL)	(mM)	1.08 (0.02) ^C^	1.05 (0.02) ^C^	0.95 (0.02) ^A,B^	15.10	2/162	<0.001
Low-density lipoprotein	(mM)	3.16 (0.07) ^B,C^	3.45 (0.07) ^A,C^	3.86 (0.07) ^A,B^	25.38	2/162	<0.001
Castelli Risk index 1 (TC/HDLc)		4.48 (0.12) ^B,C^	5.09 (0.12) ^A,C^	6.03 (0.12) ^A,B^	42.03	2/162	<0.001
AIP		1.10 (0.06) ^B,C^	1.55 (0.06) ^A,C^	1.82 (0.06) ^A,B^	42.11	2/162	<0.001
Glucose	(mM)	5.23 (0.06) ^B,C^	5.59 (0.06) ^A,C^	6.10 (0.07) ^A,B^	47.68	2/162	<0.001
Insulin	(pM)	58.68 (2.58) ^C^	65.34 (2.69) ^C^	76.61 (2.78) ^A,B^	11.30	2/162	<0.001
HOMA–2 insulin resistance index		1.11 (0.05) ^B,C^	1.25 (0.05) ^A,C^	1.49 (0.05) ^A,B^	14.17	2/162	<0.001

Results of univariate GLM analysis. All data are expressed as mean (standard error). A, B, C: results of pairwise group comparisons. DO1: indoleamine 2, 3-dioxygenase 1, AIP: atherogenic index of plasma (Log(TG/HDLc))

### Results of binary logistic regression analysis

To delineate the best predictors of NAFLD, we performed binary logistic regression analyses with NAFLD as the dependent variable and the control group as the reference group using an automatic stepwise method with all biomarkers as explanatory variables while allowing for the effects of age, sex, BMI, and TUD (Table 3). The first regression shows that the increase in zALP + zAST + zALT, triglycerides, glucose, insulin, and IDO1, coupled with a decrease in the zTRP + zKYN + z3HK + zKA score, substantially discriminated the two study groups (χ^2^ = 169.403, df = 6, *p* < 0.001). The Nagelkerke effect size was 0.872, and the classification precision was 91.7%, with a sensitivity of 88.3% and a specificity of 93.5%.

**Table 3.** Results of Binary Logistic Regression Analyses with Non-Alcoholic Liver Disease (NAFLD) or Grade 2 NAFLD Study Samples as Dependent Variables and Biomarkers as Explanatory Variables Table 3. long description.

Dichotomies	Explanatory variables	B	SE	Wald	df	*p*	OR	95% CI
1. NAFLD (vs. controls)	zALP + zAST + zALT	2.614	0.689	14.39	1	<0.001	13.660	3.538/52.735
	Triglycerides	3.097	0.827	14.04	1	<0.001	22.141	4.380/111.921
	Glucose	1.900	0.583	10.63	1	0.001	6.684	2.134/20.940
	Insulin	1.397	0.521	7.19	1	0.007	4.042	1.456/11.222
	zTRP + zKYN + z3HK + zKA	−1.252	0.488	6.58	1	0.010	0.286	0.110/0.744
	IDO1	1.673	0.601	7.75	1	0.005	5.329	1.641/17.304
2. Grade 2 (vs. grade 1)	HDL	−0.843	0.294	8.22	1	0.004	0.430	0.242/0.766
	Glucose	1.034	0.311	11.05	1	0.001	2.811	1.528/5.171
	zTRP + zKYN + z3HK + zKA	−1.394	0.372	14.07	1	<0.001	0.248	0.120/0.514

Abbreviation: ALP, alkaline phosphatase; ALT, alanine aminotransferase; AST, aspartate transferase; IDO1, indoleamine 2, 3-dioxygenase 1; 3HK, 3-hydroxy-kynurenine; HDL, high-density lipoprotein; KA, kynurenic acid; KYN, kynurenine; and TRP, tryptophan.

The second regression examined discrimination between grade 2 (dependent variable) and grade 1 (the reference group). The results show that grade 2 was best predicted by decreased HDLc and zTRP + zKYN + z3HK + zKA scores and increased glucose levels (*χ*^2^ = 52.956, df = 3, *p* < 0.001) with an effect size of 0.517 (Nagelkerke *R*^2^) and overall accuracy of 74.1%, with a sensitivity of 76.8% and specificity of 71.2%.

### Results of neural networks

**Figure 1.**
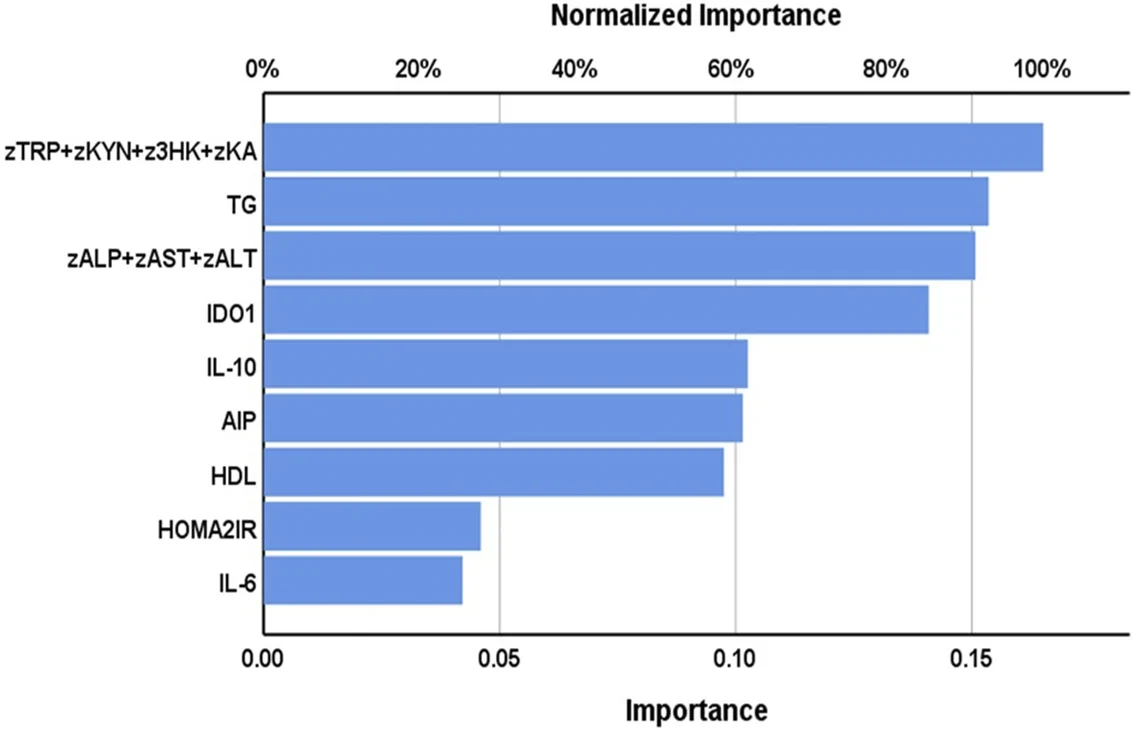
Results of neural network analysis. Importance of the biomarkers for the prediction of nonalcoholic fatty liver disease (grade 1+ grade 2) versus healthy controls. Figure 1. long description.

As shown in Table 4, the first neural network analysis (NN#1) was used to distinguish between individuals with NAFLD versus healthy controls. The model used two hidden layers with 6 units in layer 1 and 5 in layer 2. There were 2 units in the output layer. The hyperbolic tangent was used as the activation function, with an identity function in the output layer. The sum of squares was used as an error term in this neural network. The testing set had a significantly smaller sum of squares than the training set, while the incorrect prediction rate was quite constant among the three samples. This indicates that the neural network model was able to generalize the trend. The NN#1 model showed an AUC-ROC curve of 0.964 and an 89.5% accuracy of the holdout sample. Figure 1 shows the importance of the input variables. The top 4 most important predictors were the zTRP + zKYN + z3HK + zKA pathway score, triglycerides, zALT + zAST + zALT score, and IDO1, followed at a distance by IL-10, AIP, and HDLc.

**Table 4.** Results of Neural Networks with Non-Alcoholic Liver Disease or Grade 2 as Output Variables Table 4. long description.

	Models	NN#1	NN#2
Input layer	Number of units	9	9
	Rescaling method	Normalized	Normalized
Hidden layers	Number of hidden layers	2	2
	Number of units in hidden layer 1/layer 2	6/5	4/3
	Activation function	Hyperbolic tangent	Hyperbolic tangent
Output layer	Dependent variables	NAFLD (vs. HC)	Grade 2 (vs. Grade 1)
	Number of units	2	2
	Activation function	Identity	Identity
	Error function	Sum of squares	Sum of squares
Training	Sum of squares error term	6.682	8.151
	% Incorrect predictions	10.8%	24.0%
	Prediction (specificity, sensitivity)	84.0%, 89.2%	78.6%, 72.7%
Testing	Sum of squares error	2.238	3.543
	% Incorrect predictions	10.7%	21.1%
	Prediction (specificity, sensitivity)	75.0%, 95.0%	88.9%, 70.0%
	AUC ROC	0.964	0.840
Holdout	% Incorrect predictions	10.5%	20.5%
	Prediction (specificity, sensitivity)	81.5%, 96.7%	84.2%, 75.0%

AUC ROC: area under the curve of the receiver operating curve.

**Figure 2.**
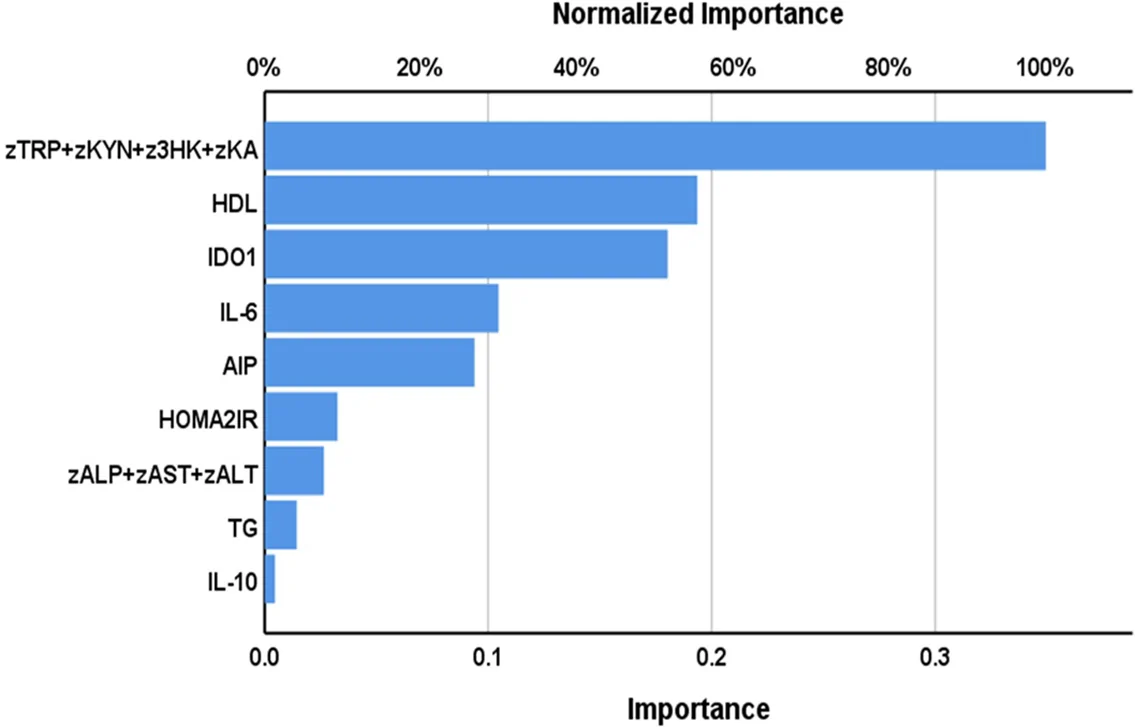
Results of neural network analysis. Importance of the biomarkers for the prediction of nonalcoholic liver disease grade 2 versus grade 1. Figure 2 long description.

The second neural network examined the discrimination between NAFLD grade 2 and grade 1. The sum of squares in the testing set was significantly lower than that in the training set. The relative error was fairly constant among the three subsets, indicating the neural network model learned to generalize from the trend. The model’s predictive accuracy was 79.5% with an AUC of 0.840. Figure 2 shows the importance and relative importance of the biomarkers predicting grade 2 versus grade 1. The most important determinant of the model’s predictive power was the zTRP + zKYN + z3HK + zKA pathway score, followed at a distance by HDLc and IDO1, and again at a distance by IL-6 and AIP.

### Intercorrelation matrix

The results of the correlation coefficients of the association between the neuropsychiatric scores and the most important biomarkers are presented in Table 5. All the serum biomarkers (all except IL-10) are significantly correlated with all neuropsychiatric domain scores, namely, pure depression, pure anxiety, pure FF, and the physiosomatic domain. There were no significant correlations between IL-6 levels and any of the clinical data. The zTRP + zKYN + z3HK + zKA score was significantly and inversely associated with the rating scales, while the other biomarkers were positively correlated.

**Table 5.** Correlation Matrix between the Neuropsychiatric Scores and the Measured Biomarkers in NAFLD Patients Table 5. long description.

Variables	Pure depression	Pure anxiety	Pure FF	Physiosomatic domain
zALP + zAST + zALT	0.360	0.380	0.478	0.549
zTRP + zKYN + z3HK + zKA	−0.439	−0.300	−0.416	−0.511
IDO1	0.394	0.329	0.358	0.503
Interleukin IL–10	0.241	0.140*	0.271	0.304
HOMA2IR	0.284	0.273	0.352	0.379
Castelli Risk index 1	0.359	0.304	0.290	0.461
Atherogenic index of plasma	0.453	0.357	0.376	0.494

All <0.01, except *: nonsignificant. IL-6 is nonsignificant. Abbreviations: ALP, alkaline phosphatase; ALT, alanine aminotransferase; AST, aspartate transferase; IDO1, indoleamine 2, 3-dioxygenase 1; 3HK, 3-hydroxy-kynurenine; HDL, high-density lipoprotein; HOMA2IR, HOMA-2 insulin resistance; KA, kynurenic acid; KYN, kynurenine; and TRP, tryptophan.

### Results of multivariate regression analysis

**Figure 3.**
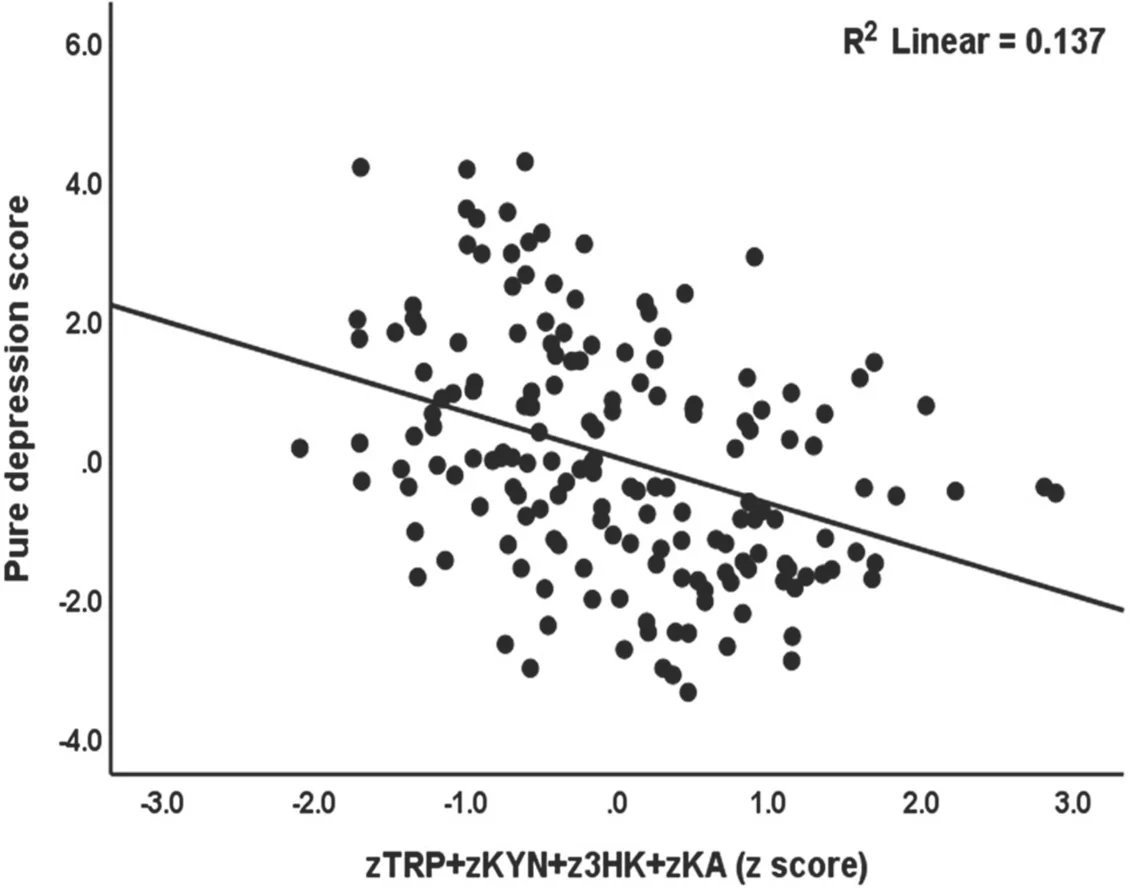
Partial regression of pure depression score in nonalcoholic fatty liver disease on an index of tryptophan and tryptophan catabolites (*p* < 0.001). TRP: tryptophan; KYN: kynurenine; 3HK: 3-OH-kynurenine; KA: kynurenic acid. Figure 3 long description.

**Figure 4.**
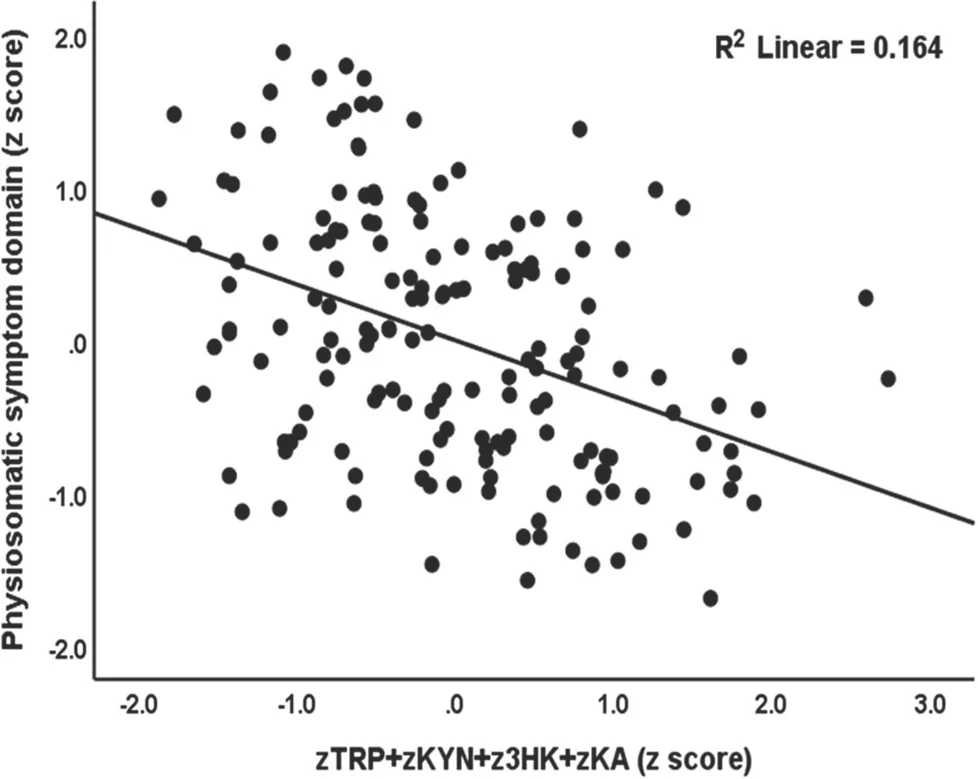
Partial regression of the physiosomatic symptom domain in nonalcoholic fatty liver disease on an index of tryptophan and tryptophan catabolites (*p* < 0.001). TRP: tryptophan; KYN: kynurenine; 3HK: 3-OH-kynurenine; KA: kynurenic acid. Figure 4 long description.

Table 6 displays the outcomes of multiple regression analyses of the neuropsychiatric rating scales on the biomarkers. Regression #1 shows that a significant part of the variance in the pure depression score (38.6%) was explained by the regression on glucose, IDO1, and AIP (all positively associated) and the zTRP + zKYN + z3HK + zKA score (negatively associated). Figure 3 shows the partial regression of pure depression score on the zTRP + zKYN + z3HK + zKA TRYCAT pathway score. Regression #2 shows that a significant part of the variance in the pure anxiety score (31.0%) was explained by the regression on glucose and the zALP + zAST + zALT index (both positively). Regression #3 shows that 39.8% of the variance in the pure FF score was explained by the zALP + zAST + zALT index, HOMA2IR, and triglycerides (all positively associated), the zTRP + zKYN + z3HK + zKA score, and the KMO index (negatively associated). Up to 60.5% of the variance in the physiosomatic domain score was explained by the cumulative effects of the zALP + zAST + zALT index, glucose, IDO1, insulin, triglycerides (all positively associated), and the zTRP + zKYN + z3HK + zKA score and KMO index (both negatively associated). Figure 4 shows the partial regression of the physiosomatic symptoms on the zTRP + zKYN + z3HK + zKA pathway index.

**Table 6.** Results of Multiple Regression with the Neuropsychiatric Scores as the Dependent Variable and All Measured Biomarkers as Explanatory Variables Table 6. long description.

Dependent variables	Explanatory variables	*β*	*t*	*p*	*F* model	df	*p*	*R*^2^
#1. Pure depression	Model				25.58	4/163	<0.001	0.386
	Glucose	0.243	3.51	0.001				
	zTRP + KYN + z3HK + zKA	−0.250	−3.73	<0.001				
	Atherogenic index of plasma	0.228	3.24	0.001				
	IDO1	0.157	2.28	0.024				
#2. Pure anxiety	Model				37.05	2/165	<0.001	0.310
	Glucose	0.421	6.29	<0.001				
	zALP + zAST + zALT	0.269	4.02	<0.001				
#3. Pure FF	Model				21.41	5/162	<0.001	0.398
	zALP + zAST + zALT	0.291	4.29	<0.001				
	zTRP + zKYN + z3HK + zKA	−0.231	−3.49	0.001				
	HOMA2-IR	0.242	3.84	<0.001				
	Triglycerides	0.184	2.76	0.006				
	KMO index	−0.134	−2.16	0.033				
#4. Physiosomatic domain	Model				31.73	8/159	<0.001	0.605
	zALP + zAST + zALT	0.249	4.32	<0.001				
	Glucose	0.225	3.94	<0.001				
	zTRP + zKYN + z3HK + zKA	−0.237	−4.29	<0.001				
	IDO1	0.174	3.03	0.003				
	Insulin	0.162	3.09	0.002				
Score	Triglycerides	0.176	3.13	0.002				
	KMO index	−0.138	−2.73	0.007				
	Triglycerides	0.188	2.87	0.005				
	Insulin	0.145	2.36	0.019				

Abbreviations: ALP, alkaline phosphatase; ALT, alanine aminotransferase; AST, aspartate transferase; IDO1, indoleamine 2, 3-dioxygenase 1; 3HK, 3-hydroxy-kynurenine; HOMA2-IR, HOMA-2 insulin resistance index; KA, kynurenic acid; KYN, kynurenine; TRP, tryptophan; and KMO index, index of kynurenine 3-monooxygenase (3HK/KYN).

## Discussion

## Metabolic characteristics of NAFLD

The primary finding of this study is that NAFLD, particularly grade 2, is characterized by metabolic abnormalities, including heightened IR, atherogenicity, liver tests, and signs of immunological activation. Prior studies indicated that liver function tests, encompassing total, direct, and indirect bilirubin, ALP, AST, and ALT, frequently yield normal results in NAFLD patients.^51^^,^^52^ However, certain NAFLD patients, particularly those with grade 2, may exhibit elevated hepatic enzymes with more pronounced steatosis.^53^^–^^55^ Consistent with other research,^56^ we observed that atherogenicity indices escalated with the advancing severity of NAFLD. Furthermore, HDLc levels were previously observed to be reduced in NAFLD as compared with the control group.^57^ Individuals with NAFLD exhibit statistically significant increases in HOMAIR levels, while IR increases with the severity of NAFLD. Furthermore, IR alone elevates the risk of NAFLD,^10^ and patients with NAFLD frequently demonstrate IR alongside low-grade inflammation.^58^

Duan et al. identified elevated inflammatory mediators, including CRP, IL-1*β*, IL-6, and TNF-*α*, as potential biomarkers in individuals with NAFLD.^59^ Consistent with other findings, our data demonstrate that modest immune activation correlates with NAFLD severity.^60^ Previous reports show that serum IL-6 levels correspond with the degree of hepatic inflammation.^61^ A prior investigation revealed elevated IL-10 levels in NAFLD compared with controls, suggesting a compensatory immunoregulatory IL-10 response aimed at mitigating proinflammatory activation.^62^ However, not all studies demonstrated elevated IL-10 levels.^59^^,^^63^^,^^64^

The liver and metabolic abnormalities in NAFLD exhibit complex and reciprocal connections. In NAFLD, atherogenic dyslipidemia is associated with heightened insulin-mediated hepatic lipid synthesis.^65^ One consequence of IR is hyperinsulinemia, which may lead to hepatic lipogenesis and fat accumulation in the liver.^9^ IR impedes insulin’s ability to inhibit hepatic glucose production, resulting in elevated circulating free fatty acids that contribute to lipid accumulation in the liver.^66^ Steatohepatitis induces hepatocellular injury when IR is coupled with a proinflammatory cytokine milieu.^67^

### Neuropsychiatric symptoms in NAFLD

The second major finding of this study is the elevation in neuropsychiatric scores, including depression, anxiety, Fibro-Fatigue, and physiosomatic domain scores in NAFLD relative to the control group, with elevated levels in grade 2 compared to grade 1. These findings align with a previous systematic review and meta-analysis indicating a greater frequency of depression, anxiety, and stress in individuals with NAFLD.^68^ Additional systematic reviews identified associations between mental health, particularly depression, and NAFLD.^20^^,^^69^ Furthermore, further data indicated a correlation between depression and the course and severity of NAFLD.^70^^–^^73^ A prior study emphasized anxiety as a significant affective symptom in NAFLD.^74^ Choi et al. indicated that elevated state and trait anxiety levels arise in accordance with the progression of liver steatosis.^21^ Chronic fatigue is common among individuals with NAFLD and may lead to adverse physical health outcomes and diminished quality of life.^75^^–^^77^

### Immune-metabolic pathways and NAFLD

The third significant finding of our investigation is that the four neuropsychiatric symptom domain scores exhibited a robust association with the cumulative effects of atherogenicity, insulin resistance, hepatic dysfunctions, and alterations in the TRYCAT pathway. Furthermore, univariate analysis revealed relationships between the majority of symptom domains and elevated IL-10 levels but not with IL-6 levels. Metabolic aberrations (liver function, atherogenicity, insulin resistance, and the TRYCAT pathway) are more significant than the immune-inflammatory indicators in predicting the symptom domains associated with NAFLD.

Major depression and anxiety disorders are associated with relative IR and a substantial correlation between the severity of IR and depressed symptoms.^78^^–^^80^ In major depression, a significant portion of the variance in overall severity of depression and anxiety was positively correlated with immune-related neurotoxicity, fasting blood glucose, insulin, and atherogenicity while inversely correlated with immune-related neuroprotection.^36^ Emerging evidence suggests correlations between atherogenicity or insulin resistance indices and the presence of neuropsychiatric symptoms in patients with major depression and medical conditions, such as Long COVID, including depressive, anxiety, chronic fatigue syndrome, and fibromyalgia symptoms.^31^^,^^36^^,^^81^^–^^83^

Consequently, we may postulate that these immune-metabolic pathways contribute to the etiology of affective and physiosomatic (including fibro-fatigue) symptoms in patients with NAFLD. IR (either peripheral or central), peripheral inflammation, neuroinflammation, and atherogenicity are components of the pathophysiology associated with NAFLD and may contribute to the onset of depression and anxiety.^19^^,^^36^ Furthermore, the inflammatory load and metabolic irregularities may contribute to the onset of chronic fatigue in patients with NAFLD.^84^ A prior study indicated that IR may significantly influence the relationship between depression and the risk of NAFLD.^85^

### TRYCAT pathway and NAFLD

The fourth significant conclusion of this study is that decreased levels of tryptophan and TRYCATs, along with elevated IDO1 concentrations, correlate with the severity of all four neuropsychiatric subdomains in patients with NAFLD. The findings on KYN and the KYN/TRY ratio contradict our initial hypothesis that inducing IDO1 activity would elevate KYN levels. In certain patients with NAFLD, the TRYCAT pathway may be stimulated by inflammatory reactions originating in the gastrointestinal tract.^39^ Prior research indicated elevated 3HK levels in NAFLD as compared with controls,^86^ while heightened IDO1 activity correlates with enhanced inflammation and fibrosis, along with elevated glucose levels, obesity, and atherosclerosis.^87^

Nonetheless, our findings indicate that tryptophan, KYN, 3HK, and KA are inversely correlated with NAFLD. Several factors may explain the associations between tryptophan/TRYACTs and the severity of neuropsychiatric symptoms in NAFLD. A shortage of tryptophan may have reduced the substrate for the TRYCAT pathway, thereby inhibiting its activity and thus the production of TRYCATs. Second, KYN generated by TDO in the liver and IDO1 in various other cells, including immune cells, is influenced by three enzymes that metabolize KYN at a rate exceeding its production, namely, kynureninase (which converts KYN into anthranilic acid), KMO (which converts KYN into 3-hydroxykynurenine), and KAT (which converts KYN into kynurenic acid). Third, discrepancies in TRYCAT levels may exist between serum and hepatic tissue. Dorochow et al. found that KYN levels are elevated in the liver but not in the bloodstream.^88^ Fourth, the protein metabolic alterations in NAFLD are intricate, involving heightened protein catabolism, the influence of insulin, and inflammatory mechanisms, together with diminished albumin synthesis.^89^ The latter is the principal tryptophan-binding protein, and its loss may further diminish the availability of tryptophan as a substrate in the TRYCAT pathway.^90^

The reduced levels of tryptophan and TRYCATs in NAFLD may have adverse effects, as the TRYCAT pathway serves as a vital anti-inflammatory and antioxidant mechanism.^38^ KYN, KA, QA, and XA inhibit IFN*γ* synthesis by activated peripheral blood mononuclear cells.^91^ KYN, KA, and XA diminish the IFNγ/IL-10 production ratio, while KA markedly diminishes the induced synthesis of TNFα,^91^ hence exerting anti-inflammatory actions.^92^ Consequently, the reduction in TRYCATs may result in diminished antioxidant activity and heightened inflammatory responses, suggesting that the disruptions in the TRYCAT pathway identified in our study could exacerbate NAFLD and its manifestations. Furthermore, KA has demonstrated neuroprotective capabilities; hence, reduced levels may contribute to heightened neurotoxicity, which is implicated in depression and anxiety.^93^

## Limitations

The findings of our study must be considered in light of their limitations. This is a case–control study; hence, no definitive causal inferences can be established. Second, the study would have been more compelling had we assessed oxidative stress parameters, including biomarkers of lipid peroxidation. Third, patients with grade 3 NAFLD were not included. Our study sought to assess serum biomarkers associated with neuropsychiatric symptoms during the early, precirrhotic stages of NAFLD (Grades 1 and 2). To maintain a pathophysiologic homogeneous cohort, we excluded Grade 3 patients, as advanced fibrosis introduces significant confounders, such as liver dysfunction, portal hypertension, and complex medication regimens that independently influence systemic inflammation and neuropsychiatric symptomatology. This approach allowed us to more precisely delineate the relationship between NAFLD-associated metabolic abnormalities and neuropsychiatric disorders prior to the onset of overt cirrhosis.

## Conclusions

The severity of depression, anxiety, and fibro-fatigue symptoms was considerably higher in NAFLD than in controls and in grade 2 compared to grade 1 NAFLD. Lowered tryptophan and TRYCAT levels are independently associated with the severity of these affective and physiosomatic symptoms in patients with NAFLD. In addition, atherogenicity and IR further aggravate the severity of the neuropsychiatric symptoms, and these metabolic disorders are more important than immune activation. The results show that the TRYCAT pathway, especially lower tryptophan, is another drug target to treat NAFDL and neuropsychiatric symptoms due to NAFDL in conjunction with care as usual. Previously, it was shown in animal models that tryptophan supplementation can improve liver function, the accumulation of lipids in the liver, immune-inflammatory responses, and increased gut permeability.^94^^,^^95^ Nevertheless, there are also reports that tryptophan injections, especially combined with high-fat diets, might cause the accumulation of fatty acids in the liver and induce NAFLD.^96^

## Data Availability

The database created during this investigation will be provided by the corresponding author (MM) upon a reasonable request once the authors have thoroughly used the data set.

## Table 1. Long description

This table presents demographic, clinical, and biochemical parameters across three groups: healthy controls (n = 60), grade 1 nonalcoholic fatty liver disease patients (n = 56), and grade 2 NAFLD patients (n = 52). Age shows no significant difference across groups (p = 0.775). Sex distribution is not significantly different. Smoking and marital status also show no significant differences. Employment status differs significantly, with more unemployment in NAFLD groups. Body mass index increases progressively from controls to grade 1 to grade 2, though not statistically significant. Depression scores increase significantly across groups: controls, grade 1, and grade 2. Anxiety scores also increase significantly: controls, grade 1, and grade 2. FibroFatigue scores increase from controls to grade 1 to grade 2. Physiosomatic scores show no significant differences. Total serum bilirubin shows no significant differences. Direct bilirubin is significantly lower in grade 1 compared to controls and grade 2. Indirect bilirubin increases progressively: controls, grade 1, and grade 2. Alkaline phosphatase (ALP) increases from controls to grade 1 and grade 2. Aspartate transferase (AST) increases from controls to grade 1 to grade 2. Alanine aminotransferase (ALT) shows no significant differences. AST/ALT ratio decreases from controls to grade 1 to grade 2, not significant. The composite z-score (zALP + zAST + zALT) increases from controls to grade 1 to grade 2. Superscript letters A, B, C indicate significant pairwise comparisons: A = significantly different from HC, B = significantly different from Grade 1, C = significantly different from Grade 2.

Navigate back to Table 1..

## Table 2. Long description

This table presents serum levels of tryptophan-kynurenine pathway metabolites, inflammatory cytokines, lipid profiles, and glycemic markers across three groups: healthy controls (n = 60), grade 1 NAFLD (n = 56), and grade 2 NAFLD (n = 52). Results are from univariate GLM analysis expressed as mean (standard error). Key findings: L-Tryptophan decreases significantly across groups: controls, grade 1, grade 2. KYN/TRP (IDO index) shows no significant difference. Triglycerides increase from controls to grade 2. HDL decreases from controls to grade 1 to grade 2. LDL increases from controls to grade 2. Castelli Risk index increases from controls to grade 2. AIP increases from controls to grade 2. Glucose and insulin also increase significantly. Superscript letters A, B, C indicate significant pairwise comparisons: A = significantly different from HC, B = significantly different from Grade 1, C = significantly different from Grade 2.

Navigate back to Table 2..

## Table 3. Long description

This table presents binary logistic regression analyses. First dichotomy: NAFLD versus healthy controls. Significant predictors include zALP + zAST + zALT, triglycerides, glucose, insulin, zTRP + zKYN + z3HK + zKA, and IDO1. Second dichotomy: Grade 2 NAFLD versus Grade 1 NAFLD. Significant predictors include HDL, glucose, and zTRP + zKYN + z3HK + zKA. All explanatory variables are listed with their B coefficients, standard errors, Wald statistics, degrees of freedom (all 1), p-values, odds ratios, and 95% confidence intervals. Abbreviations: ALP = alkaline phosphatase, ALT = alanine aminotransferase, AST = aspartate transferase, IDO1 = indoleamine 2,3-dioxygenase 1, HDL = high-density lipoprotein.

Navigate back to Table 3..

## Table 4. Long description

This table presents two neural network models. NN#1 predicts NAFLD versus healthy controls with input layer of 9 normalized units, two hidden layers (6 and 5 units) with hyperbolic tangent activation, and output layer with 2 units using identity activation and sum of squares error function. Training results: sum of squares error 6.682, 10.8% incorrect predictions, specificity 84.0%, sensitivity 89.2%. Testing results: sum of squares error 2.238, 10.7% incorrect predictions, specificity 75.0%, sensitivity 95.0%, AUC ROC 0.964. Holdout results: 10.5% incorrect predictions, specificity 81.5%, sensitivity 96.7%. NN#2 predicts Grade 2 versus Grade 1 NAFLD with input layer of 9 normalized units, two hidden layers (4 and 3 units) with hyperbolic tangent activation, and output layer with 2 units. Training results: sum of squares error 8.151, 24.0% incorrect predictions, specificity 78.6%, sensitivity 72.7%. Testing results: sum of squares error 3.543, 21.1% incorrect predictions, specificity 88.9%, sensitivity 70.0%, AUC ROC 0.840. Holdout results: 20.5% incorrect predictions, specificity 84.2%, sensitivity 75.0%.

Navigate back to Table 4..

## Figure 1. Long description

Bar chart showing normalized importance of 9 biomarkers for predicting NAFLD (grades 1 + 2) vs controls. zTRP + zKYN + z3HK + zKA ranks highest at 0.16%.

Navigate back to Figure 1..

## Figure 2 Long description

Bar chart showing normalized importance of 9 biomarkers for predicting grade 2 vs grade 1 NAFLD. zTRP + zKYN + z3HK + zKA ranks highest at 0.95%, followed by HDL at 0.50%.

Navigate back to Figure 2.

## Table 5. Long description

This table shows Pearson correlation coefficients between four neuropsychiatric outcome variables (pure depression, pure anxiety, pure FF/FibroFatigue, and physiosomatic domain) and seven explanatory biomarkers. All correlations are significant at p < 0.01 except where noted. zALP + zAST + zALT correlates with all outcomes: depression 0.360, anxiety 0.380, FF 0.478, physiosomatic 0.549. zTRP + zKYN + z3HK + zKA shows negative correlations: depression −0.439, anxiety −0.300, FF −0.416, physiosomatic −0.511. IDO1 correlates positively: depression 0.394, anxiety 0.329, FF 0.358, physiosomatic 0.503. Interleukin-10 shows weaker but significant correlations: depression 0.241, FF 0.271, physiosomatic 0.304 (anxiety 0.140 nonsignificant). HOMA2IR correlates: depression 0.284, anxiety 0.273, FF 0.352, physiosomatic 0.379. Castelli risk index 1 correlates: depression 0.359, anxiety 0.304, FF 0.290, physiosomatic 0.461. Atherogenic index of plasma correlates: depression 0.453, anxiety 0.357, FF 0.376, physiosomatic 0.494. IL-6 was nonsignificant and not shown. Abbreviations: ALP = alkaline phosphatase, ALT = alanine aminotransferase, AST = aspartate transferase, IDO1 = indoleamine 2,3-dioxygenase 1, HDL = high-density lipoprotein, HOMA2IR = HOMA-2 insulin resistance index.

Navigate back to Table 5..

## Table 6. Long description

This table presents four separate multiple regression models. Model 1 (pure depression): significant predictors include glucose, zTRP + zKYN + z3HK + zKA, atherogenic index of plasma, and IDO1. Model 2 (pure anxiety): significant predictors are glucose and zALP + zAST + zALT. Model 3 (Pure FF/FibroFatigue): significant predictors include zALP + zAST + zALT, zTRP + zKYN + z3HK + zKA, HOMA2-IR, triglycerides, and KMO index. Model 4 (physiosomatic domain score): significant predictors include zALP + zAST + zALT, glucose, zTRP + zKYN + z3HK + zKA, IDO1, insulin, triglycerides, KMO index. Each model includes F-statistic, degrees of freedom, p-value for the model, and R2. Abbreviations: ALP = alkaline phosphatase, ALT = alanine aminotransferase, AST = aspartate transferase, IDO1 = indoleamine 2,3-dioxygenase 1, HOMA2-IR = HOMA-2 insulin resistance index, KMO index = kynurenine 3-monooxygenase index (3HK/KYN).

Navigate back to Table 6..

## Figure 3 Long description

Scatter plot displaying a strong negative linear relationship between the composite tryptophan catabolite index (zTRP + zKYN + z3HK + zKA) on the X-axis ranging from approximately −2.8 to +3.0 and pure depression score on the Y-axis ranging from approximately −14.2 to +1.0. Each point represents an individual subject with nonalcoholic fatty liver disease. A downward-sloping regression line indicates that higher tryptophan catabolite index values (more positive z-scores) are associated with lower (more negative) depression scores. The data points cluster relatively tightly around the regression line, suggesting a moderately strong inverse correlation. Statistical significance is reported as p < 0.001. Abbreviations: TRP = tryptophan, KYN = kynurenine, 3HK = 3-hydroxykynurenine, KA = kynurenic acid.

Navigate back to Figure 3.

## Figure 4 Long description

Scatter plot displaying a negative linear relationship between the composite tryptophan catabolite index (zTRP + zKYN + z3HK + zKA) on the X-axis, ranging from approximately −1.8 to +3.0, and the physiosomatic symptom domain Z-score on the Y-axis, ranging from approximately −3.9 to +0.9. Each point represents an individual subject with nonalcoholic fatty liver disease. A downward-sloping regression line indicates that higher tryptophan catabolite index values (more positive z-scores) are associated with lower (more negative) physiosomatic symptom scores. The data points show moderate scatter around the regression line, suggesting a consistent but not extremely strong inverse correlation. Statistical significance is reported as p < 0.001. Abbreviations: TRP = tryptophan, KYN = kynurenine, 3HK = 3-hydroxykynurenine, KA = kynurenic acid.

Navigate back to Figure 4.
